# Integration of early identification and brief intervention in frontline health services: the case of Quebec

**DOI:** 10.1186/1940-0640-7-S1-A74

**Published:** 2012-10-09

**Authors:** Marianne Saint-Jacques, Thomas G Brown, Sarah Filion-Bilodeau, John Topp, David Ross, Lucie Legault

**Affiliations:** 1 Department of Medicine and Health Sciences, University of Sherbrooke, Longueuil, Quebec, Canada; 2 Addiction Research Center, McGill University, Montreal, Quebec, Canada; 3 Douglas Mental Health University Institute, McGill University, Montreal, Quebec, Canada; 4 Addiction Services, Pavillon Foster Addiction Treatment Center, St. Philippe de Laprairie, Canada; 5 Addiction Research Program, Douglas Mental Health University Institute, Montreal, Quebec, Canada

## 

In 2006, the Quebec provincial government committed itself to developing capacity for evidence-based alcohol and other drug screening and brief intervention (SBI) in frontline health-service delivery settings. Two studies were conducted prior to province-wide deployment to inform decision makers as to possible implementation challenges. Study 1 evaluated the efficacy of implementing a model of SBI, adapted from the World Health Organization, in three frontline public health services. Sixty-two clinicians were trained in systematic detection of substance use and brief motivational intervention. Chart review of new admissions (N = 453) prior and following training assessed clinician conformity to SBI guidelines. Forty percent of patients endorsed at least one item on the four-question CAGE-AID (Cut Down, Annoyed, Guilty, Eye Opener screen Adapted to Include Drugs). Systematic screening among new admissions peaked at 70% and decreased to pre-training levels seven months after training. However, more than half of all new patients were assessed with validated questionnaires rather than informal questioning. Implementation of motivational interviewing techniques led to modest changes in clinician interventions. A second qualitative study was conducted to document possible organizational barriers to SBI implementation. Interviews were conducted with 24 clinicians and program administrators from frontline services of the six main health regions of Quebec province. Choice of vocabulary used in public policy documents and program material and sequence of delivery of services created confusion among clinicians as to what constituted alcohol and other drug SBI. Many resources developed for other SBI health programs in these frontline services seemed to be transferable to alcohol and drug SBI. Lessons learned were as follows: complexity in the SBI program is inversely related to the subsequent integrity of program deployment; clarity as to what constitutes SBI is paramount; and integration of parallel competencies/related programs could facilitate deployment of alcohol and other drug SBI.

